# TIME, to move forward? comment on “a universal outcome measure for headache treatments, care-delivery systems and economic analysis”

**DOI:** 10.1186/s10194-022-01395-y

**Published:** 2022-02-22

**Authors:** Raquel Gil-Gouveia

**Affiliations:** 1 Present Address: Hospital da Luz Headache Center, Lisboa. Avenida Lusíada nº 100, 1500-650 Lisboa, Portugal; 2 Universidade Católica Portuguesa, Institute of Health Sciences, Center for Interdisciplinary Research in Health, Lisboa, Portugal

## Abstract

The paper from Steiner et al. suggests that an outcome measure expressed in time units may be an adequate method to assess the impact of headache disorders, regardless of diagnosis or health care setting, proving useful for cost-benefit analysis and health policy definition. Using time lost to each attack – weighted by disability – may prove to be a reliable measure to establish the effectiveness of acute treatment, but if considering also the attack frequency it could evaluate the effects of preventive strategies. A measure such as the Headache Gauge, which translates the proportion of time lost to headache -related disability, has proven to be applicable also in routine clinical practice as well, and can be tested in clinical trials and populational analysis. There are practical limitations, such as disability assessment and the need for prospective data collection to avoid recall bias but it seems consensual that impairment related to primary headache disorders is primarily driven by the TIME stolen from the perfect health status.

## Main Body

There is a huge unmet need to quantify the impact of headache disorders on those who suffer, as no single outcome measure is capable of expressing the full dynamics of headache-related impairment. Many variables need to be accounted for in such an effort, whether deriving from attack-related disability (which varies between and within attacks, depends on the coping mechanisms and planned tasks, can be attributed to different symptoms within each headache disorder and between different diagnosis, and it is clearly *not* a surrogate of pain intensity [1]), from attack frequency and duration [2] and, of course, interictal impact [3]. Adding to the complexity of providing a reliable measure, it also needs to be simple, understandable, cross-cutting and useful in different contexts - clinical trials, economic modeling and real-life clinical environment with different cultural and linguistic backgrounds.

There is, however, “universal outcome measure” that is obviously presented in the article by Steiner et al. [4], which is TIME. Time lost to attacks – or hours lost to (or lived with) disability (HLDs) [4] - whether accounted for by single attacks when evaluating acute treatments or computed by life-periods when evaluating preventive interventions, is a surrogate that is easily understandable and intuitive. It is also independent of context, such as diagnosis, setting or cultural background. In a previous effort to derive such a measure for clinical use in primary headaches, we compared patients with migraine and tension-type headache and found that the main factor of disability - as accessed - was time spent within attacks [5].

Two main limitations to this approach persist. First, not all time spent on an attack has the same degree of disability. Secondly, data maybe unreliable, if not collected prospectively.

As for the first barrier - assessment of disability - Steiner et al suggests using the definition of Weight of Disability (DW) from the Global Burden of Disease (GBD) [6], to avoid the subjective retrospective assessment of attacks, but it also does not consider time spent in each disability state. This is a valuable practical strategy for population-based and likely economic studies, as it is based on a comprehensive estimate conveyed by a large-scale survey of general population judgments about health losses associated with many causes of illness and injury [6]. However, it is hardly suitable for clinical trials or real-life scenarios, as the variability of disability needs to be considered. Although arbitrary, the definition of disability on a verbal rating ordinal scale (VRoS) may be most adequate solution. It is advisable for the international scientific community to argue and validate the best definition of scale for this purpose, but we have found that a down-to-earth 4-note scale is easily understandable and practical. An example: (1) to be able to fully function within the attack; (2) attack that interferes with normal activities; (3) attack that prevents normal activities and (4) attack that precludes all activities, resulting in being bedridden or hospitalized) [5].

Using this ordinal disability scale allows you to estimate the hours lost due to the disability by multiplying the time (either absolute time or also in a 4-note VRoS) by the disability of each attack resulting in a daily impact score (*s*_*i*_), which can be used for acute care assessments. In the case of evaluating preventive measures, simply multiply each daily impact score(*s*_*i*_) by the frequency of its occurrence (*n*_*i*_), in a given period of time (N) and obtain a weighted average that translates the proportion of time spent with a disability in any chosen period – the “headache gauge” (HG), $HG=\frac{\sum s_in_i}{16N}\times100$ [5].

The second barrier more difficult to overcome, as accuracy relies on prospectively collecting data to avoid memory or motivation biases [7, 8]. As a proof-of-concept exercise, we re-analyzed the headache gauge validation database and calculated an alternative gauge at inclusion, multiplying the perceived mean headache frequency, mean attack duration and mean attack disability, in the previous month, reported by memory, only in patients using headache calendars (*N*=80) irrespective of headache diagnosis. The HG scores distribution for both variables were not normal, but rather right-skewed gamma distributions. The average actual HG score for this sample was 9.47 (sd ± 7.54) with both a median and mode of 6.67, ranging from 0.63 to 33.96 while the alternative HG score average was 8.93 (sd ± 9.84), with a median of 5.47 and a mode of 2.40, ranging from 0.13 to 48.00. Both distributions were strongly correlated (Spearman 0.831, *p* < 0.0001, two-tailed) although data derived from perceived average impact had higher variance (96.81 versus 56.78), positive skewness (1.82 versus 1.54) and kurtosis (3.38 versus 2.09), so dispersion was higher.

If the same analysis is plotted only with those patients at inclusion *not* presenting with diaries, in which the information from the last month was obtained by memory (*N*=148) to calculate both scores, the average HG score (information retrieved by remembering attack-by-attack) was 11.54 (sd ± 10.75, median 7.95, mode of 3.75, range 0.21 to 58.33) while the alternative HG score averaged 13.85 (sd ± 15.77, median 8.00, mode 9.60, range 0.05 to 79.20); again showing good correlation (Spearman 0.740, *p*< 0.0001, two-tailed) but dispersion was much higher in both, resulting in a very high variance (248.80 versus 115.52), positive skewness (1.85 versus 1.76) and kurtosis (3.52 versus 3.11).

These data support that the more we depend on obtaining data from memory, even considering only a relatively short period of time (such as the previous month) the greater will be the variability of the obtained data, resulting in less precision. Obtaining patient data is always a challenge, but since there is no wearable attack meter available *yet*, we need to rely on patient reports. So far, diaries seem to be the most reliable instruments [8] so time-related plotted data such as the headache gauge, which can be automatically retrieved from a simple universal web-based diary or calculated from a paper diary, would be the most reliable source of information for any assessment purpose, whether clinical or populational.

The question remains, when deciding on a universal outcome measure for reporting disability in headache disorders, is it TIME, to move forward?

## References

[1] Gil-Gouveia R, Oliveira AG, Martins IP (2015). The impact of cognitive symptoms on migraine attack-related disability. Cephalalgia.

[2] Lipton RB, Manack Adams A, Buse DC, Fanning KM, Reed ML (2016). A Comparison of the Chronic Migraine Epidemiology and Outcomes (CaMEO) Study and American Migraine Prevalence and Prevention (AMPP) Study: Demographics and Headache-Related Disability. Headache.

[3] Lampl C, Thomas H, Stovner LJ, Tassorelli C, Katsarava Z, Laínez JM (2016). Interictal burden attributable to episodic headache: findings from the Eurolight project. J Headache Pain.

[4] Steiner TJ, Linde M, Schnell-Inderst P (2021). A universal outcome measure for headache treatments, care-delivery systems and economic analysis. J Headache Pain.

[5] Gil-Gouveia R, Marques IB, Parreira EP, Martins IP, Oliveira AG (2021). Headache Gauge: a real-life calendar-based tool for headache monitoring. Neurol Sci.

[6] WHO. WHO methods and data sources for global burden of disease estimates 2000-2011. 2013;(November):86. Available from: http://www.who.int/healthinfo/statistics/GlobalDALYmethods_2000_2011.pdf?ua=1

[7] McKenzie JA, Cutrer FM (2009). How well do headache patients remember? A comparison of self-report measures of headache frequency and severity in patients with migraine. Headache.

[8] Gil-Gouveia R, Oliveira AG. Are PROMs passing the message? A reflection with real-life migraine patients. Cephalalgia. 2022;42(2):162-165. 10.1177/03331024211034509. 10.1177/03331024211034509 34407643

